# A repeated awakening study exploring the capacity of complexity measures to capture dreaming during propofol sedation

**DOI:** 10.1038/s41598-025-12695-z

**Published:** 2025-09-24

**Authors:** Imad J. Bajwa, Andre S. Nilsen, René Skukies, Arnfinn Aamodt, Gernot Ernst, Johan F. Storm, Bjørn E. Juel

**Affiliations:** 1 Brain Signaling Group, Division of Physiology, Department of Molecular Medicine, Institute of Basic Medical Sciences, Faculty of Medicine, University of Oslo, 0317 Oslo, Norway; 2 Vestre Viken Klinisk Nevrovitenskap, Kongsberg Hospital, 3612 Kongsberg, Norway; 3 Stuttgart Center for Simulation Science, University Stuttgart, 70174 Stuttgart, Germany

**Keywords:** Consciousness, Neurophysiology, Neuroscience, Physiology

## Abstract

Patients undergoing general anesthesia are often assumed to be unconscious. However, it is known that conscious experiences in the form of dreams occur even in unresponsive states induced by anesthetics. Here, we recorded resting state electroencephalography (EEG) as well as EEG combined with TMS perturbations in 20 healthy participants during propofol sedation. Participants were repeatedly awoken from deep sedation and asked immediately whether they had experiences just before waking up and what they experienced. Out of the 52 attempted awakenings in this study, 24 produced reports of having had an experience, while there were 5 reports of no experience. In the remaining 23 attempts, the subject was either unarousable or the report too incoherent to provide information about their experience prior to awakening. We then tested whether two different consciousness measures based on EEG complexity—the state transitions perturbational complexity index (PCI^st^) and single-channel Lempel-Ziv complexity (LZc)—differed between awakenings with and without experience. While our study confirms earlier findings that the EEG complexity measures significantly decrease from the awake state to the sedated state, we find no evidence that these measures differ between periods associated with reports of dreaming and non-dreaming, within the sedated state. A few interpretations and limitations are discussed.

**Supplementary Information:**

The online version contains supplementary material available at 10.1038/s41598-025-12695-z.

## Introduction

So far, there is no established method to objectively measure a person’s state of consciousness (here defined as any subjective experience, including dreams and any other experiences disconnected from the environment). Establishing such a method has major implications for clinical and basic research purposes^1^. Currently, clinical assessments of the level of consciousness in patients rely on their ability to interact with their external environment and give reports of their subjective experiences^2^. In some cases, however, such as in ketamine anesthesia, sleep, or disconnected states like the “locked in syndrome,” patients can have conscious experiences without the ability to report them^3–6^. Therefore, it is important to find objective measures that can identify consciousness independently of the subject’s ability to interact with their environment^7^.

Consciousness has long been considered related to complex neural activity^8^ and numerous studies have emphasized its importance as key indicators of consciousness^9,10^. This perspective is echoed in several theoretical frameworks, such as the integrated information theory (IIT)^11^ and Global Neuronal Workspace Theory (GNWT)^12^. Systems that fulfill the criteria of being highly integrated and differentiated are thought to exhibit dynamics with a high degree of complexity, a notion that can be quantified using a variety of mathematical tools. Some of the most promising tools that exist to date are based on the complexity of cortical dynamics^9^. Two such measures are the Lempel-Ziv perturbational complexity index (PCI^LZ^)^10^ which calculates the complexity of an evoked response to transcranial magnetic stimulation (TMS)—and its generalization to sensor space, PCI state transition (PCI^st^)^13^ —and Lempel-Ziv complexity of the spontaneous EEG signal (LZc)^14^.

These measures of complexity have already been applied to recordings of brain activity and have been successfully used as indicators of conscious state in some cases^13–15^. Typically, this has been done by investigating how the measures change between conscious and seemingly unconscious states, such as wakefulness and general anesthesia^9^. Fewer studies have, however, investigated whether these measures differ between the presence or absence of conscious experiences in what appears to be the same physiological state (but see for example Aamodt et al. and Nieminen et al.^16,17^). This is important because people are known to report conscious experiences in states where they appear unconscious, for example during general anesthesia and deep sleep, if they are asked to describe their experiences immediately upon awakening^7,16,18–20^. A few studies have contrasted properties of spontaneous and evoked EEG between conscious and unconscious states within the same physiological state—in both non-REM sleep^7,16,18,20^ and propofol anesthesia^21^. However, as far as we know no study to date has investigated how complexity measures of consciousness, specifically, vary with the presence of experience during sedation using an intermittent awakening paradigm.

Here we report the results of a study with repeated awakenings from deep propofol sedation, taking the subjective reports of dreams as an indicator of whether the participant had a dream experience before the awakening. Thus, extrapolating from theory, we tested the hypothesis that:

1. PCI^st^ and single-channel LZc differ between awakenings with and without reports of dream experience, within the same level of sedation.

Additionally, in line with previous studies, we hypothesized that:

2. PCI^st^ and single channel LZc will be higher in wakefulness than the sedated state.
3. Single-channel LZc will be higher with eyes open than eyes closed conditions during wakefulness.

These hypotheses rely on two important assumptions. First, that the sedation state is reasonably stable throughout the measurements, and secondly that the associated state of (un)consciousness is stable (i.e. that the degree of consciousness or probability of having experiences) within the same period.

## Methods

### Participants

Healthy volunteers were recruited through online social media advertising and posters distributed at the University of Oslo. Participants were screened for contraindications to magnetic resonance imaging (MRI) and transcranial magnetic stimulation (TMS) according to local hospital guidelines and safety precautions developed by Rossi, et al.^22^ respectively. A total of 20 participants (median age 26 [range 19–30] years, 12 female) were invited to complete the sedation protocol (see “Study design”). Prior to the sedation protocol, participants fasted from solid foods for > 6 h, and from fluids for > 2 h. Participants were financially compensated. The study was approved by the Ethics Committee REK (2015/1520) and written consent was obtained from all participants after being thoroughly informed about the study. All methods were performed in accordance with relevant guidelines and regulations and the Declaration of Helsinki.

### Study design

Before the sedation experiment, participants underwent structural MRI to obtain a 3D anatomical model of their head and brain. This was later used to provide reliable and accurate anatomical navigation in the application of TMS during the experiment. Participants were then tested for TMS compatibility (likelihood of high signal to noise ratio) by approximating their motor threshold (MT) (stimulation intensity at which 50% of TMS pulses over the optimal spot in primary motor cortex generate twitches in the pollicis brevis—thumb— muscle as recorded with surface electromyography^23^) and stimulating their scalp near the vertex to look for artefactual muscle twitches with an intensity of 130% of the MT. This quick assessment was performed visually prior to MRI. This is because if MT is easy to estimate and TMS does not lead to muscle activation in the scalp or face, then it is more likely we will get a good signal to noise ratio with the inclusion of the MRI to guide targeting and algorithmic estimation of the RT. Participants with visible muscle twitches in the face, neck or scalp produced by TMS to both frontoparietal and parietooccipital areas, or who had an estimated MT over 80% of maximum stimulation capacity of the TMS equipment, were excluded from the study. From the 20 participants included, 3 participants did not complete the TMS part of the study due to either having too high a motor threshold on the day of the experiment, having pronounced muscle twitches during or prior to the data acquisition, or because a suitable target for stimulation could not be found on the day of the sedation (insufficient signal to noise ratio). From a total of 55 TMS-EEG recordings (including wakefulness and up to three sedation recordings pr. participant), 4 were excluded after preprocessing due to low signal-to-noise ratio in the TMS evoked response, prior to computing PCI^st^.

On the day of the sedation protocol, we estimated the participant’s MT using an automatic algorithm^24^ and recorded EEG from each participant during wakefulness (as assessed by Richmond agitation sedation scale (RASS)^25^ : 0). Resting state awake EEG was recorded for 5 min with eyes open and 5 min with eyes closed, and TMS-EEG with eyes closed (up to 300 single pulses to a parieto-occipital or frontal region, at an intensity > 120% of MT, with an average inter-pulse interval of 2 ± 0.3 s). Following the TMS protocol, participants were asked whether they had an experience immediately prior to being prompted, and to specify the contents of their experience (for a detailed view of questionnaires, see Tables 1 and 2 in the main text and Table S1 in the supplementary materials).

Following the wakefulness conditions, propofol was administered by a licensed anesthesiologist (in accordance with national hospital safety guidelines). The participants were instructed to slowly count upwards from 1, while infusion rate was increased in a stepwise manner until the subject stopped counting and fell asleep, without the use of a bolus dose for induction. The level of propofol sedation was titrated towards a target level of deep sedation (RASS: -4), in which it was possible to partly wake up (arouse) the participants by strong stimulation (pinching of their trapezius muscle and loudly calling the participant’s name) in order to permit verbal communication with the subject. This resulted in individualized maintenance dosages (median: 2.0, range 0.8–3 µg/ml) as the effect of propofol can vary considerably between individuals^26^. After the participant had fallen asleep, the sedation level was investigated by an attempted awakening every 5 min and adjusted accordingly until RASS: -4 was achieved. Once the correct, individualized dosage was found, participants were asked to relax with their eyes closed and were allowed to rest in the sedated state for at least 5 min before the beginning of measurements.

As with wakefulness, we recorded TMS-EEG (same stimulation intensity and target as wakefulness recordings), followed by 1-minute resting state EEG (eyes-closed) just before they were woken up and questioned about their experiences (see Tables 1 and 2 for questionnaires). This paradigm was repeated up to three times for each participant, but no participant was kept in the sedated state for more than three hours. After the last awakening, participants were allowed to fall back asleep before the propofol infusion was stopped. All verbal reports from the participants were cross-checked with video and audio taken during the sessions.

### Data acquisition

EEG/TMS-EEG data was recorded using multi-channel BrainAmp amplifiers with the BrainVision Recorder software (version 1.25.0204, BrainProducts GmbH, Gilching Germany, https://www.brainproducts.com) with a sampling frequency of 5000 Hz. 62 passive EEG electrodes, and an additional 2 electrooculogram (EOG) channels, were placed according to the international 10–10 system (EasyCap, BrainProducts GmbH, Gilching Germany). The reference electrode was placed 2 cm above the nasion, and the ground electrode was placed above the right eyebrow. Due to the length of the experiment, impedances were inspected before each recording with a target to keep them below 10 kΩ, and electrodes were adjusted (e.g. cleaned or re-gelled) as needed.

TMS pulses were applied to either frontal (approximately Brodmann Areas: 6) or parieto-occipital (approximately BA 7 or BA 5) regions of the dominant hemisphere based on evoked responses achieved prior to sedation. The PowerMag Research 100 stimulator was used in conjunction with a figure-eight coil for TMS stimulation (Double coil PMD70-pCool, MAG & More GmbH, München, Germany). To ensure reliable and repeatable positioning of the stimulation coil, we used T1 weighted MRI images (Philips 3.0T Ingenia MR system, Philips Healthcare, Netherlands). The position of TMS coil and the participant’s head in a 3D space was tracked using the PowerMag View! (version 2.0.7.771, MAG & More GmbH, München, Germany, https://magandmore.com/) software. During registration of EEG and TMS-EEG, participants wore earplugs with a noise-masking sound based on phase shuffled samples of the TMS pulse to reduce auditory TMS-evoked potentials.

Following recording of EEG/TMS-EEG data, participants were awoken by loudly calling their name while firmly squeezing their right or left trapezius muscle. Their immediate reports about experience prior to awakening were then recorded and categorized based on responses to questions 1 and 2 of the questionnaire (see Tables 1 and 2 below).

### Preprocessing

#### Spontanuous EEG

EEG/TMS-EEG data was analyzed using Python and the MNE-Python package^27^ along with custom scripts (available on Github: https://github.com/SugarLab/Anesthesia_PCI_Cont/). The spontaneous EEG data was preprocessed as follows: down sampled to 1000 Hz, applied a bandpass FIR-filter at 0.5–40 Hz, set EEG reference to frontal reference channel, and removed eye artifacts using linear regression based on EOG channels with the EOG regression function from the MNE-Python package^27,28^. We then epoched the data into 5-second-long epochs and applied the AutoReject algorithm^29^. This algorithm works by using Bayesian optimization to estimate optimal peak-to-peak thresholds for each channel to remove epochs. Then, depending on whether a significant number of channels identify an epoch as bad it is rejected, or in the case where trials are bad in only a few channels, the channels are interpolated rather than rejecting whole epochs. On average, over awake spontaneous recordings the AutoReject algorithm interpolated 9% (range = 3–17%) and rejected 4% (range = 0–16%) of the segments (here defined as one epoch of a single EEG channel). A further 3% of all epochs (range = 0–49%) were rejected. From the 1-minute spontaneous recordings, the AutoReject algorithm interpolated 20% (range = 2–36%) and rejected 6% (range = 0–30%) of the segments (here defined as one epoch of a single EEG channel). A further 8% of all epochs (range = 0–42%) were rejected. The total length of the 60-second EEG recordings were reduced to a median of 55 s (range: 35–60 s). Finally, we applied an average reference and independent component analysis (ICA) with 20 components was computed. Individual components were inspected by eye based on PSD plots, topography, and variance across epochs. Artefactual components resembling noise, pulse artifacts, eye movements, or muscle twitches were removed. Across EEG recordings, a median of 0 (range = 0–8) components for the awake and 0 (range 0–1) for the 1-minute sedation were removed per recording.

The spontaneous EEG data was used to calculate single-channel LZc using code from the pyconscious toolbox^30^.

The computation of single-channel LZc is based on the analytical representation of the EEG signal. The analytical representation of the EEG signal is a complex-valued time series, which can be viewed as the sum of one real component (the EEG signal itself) and one imaginary component (the Hilbert transform of the EEG signal). Alternatively, this analytical representation can be written as the pointwise product of a time-varying real-valued positive amplitude and a complex unit vector with a time-varying phase angle.

Single-channel LZc was then calculated by binarizing the amplitude (absolute value) of the analytical representation of the EEG signal, thresholding on its median value within each calculation window. A Lempel-Ziv compression algorithm was applied to obtain a list of “binary words” that appear at least once in the data matrix. The single channel LZc for the particular channel is given by the number of binary words normalized by the comparable value obtained for the same binary input sequence randomly shuffled. Single channel LZc was computed by taking the average of 5-second epoch values across the full recording and finally taking the average LZc value across all channels to obtain one LZc value per recording. Thus, each LZc value quantifies the complexity of spontaneous EEG over (up to) 5 min in the awake conditions, and 1 min in the sedation condition. LZc was normalized using a permutation-based method from the pyconcious toolbox that randomly shuffles the binarized data sequence once (maintaining the equal distribution of 1s and 0s) to generate a comparison sequence which the data is normalized to.

#### Evoked EEG

For the TMS-EEG data we first epoched the data from − 250 ms pre-pulse to 500 ms post-pulse onset. To remove the TMS pulse artifact, linear interpolation was employed using the MNE function “fix_stim_artifact”^27^ spanning from − 2 ms to an individually visually selected endpoint for each recording (median 7ms [range: 4-16ms]). Bad channels were marked by visual inspection with a median of 7 channels (range: 2–26) being interpolated.

Subsequent steps included resampling the data to 1000 Hz and applying a bandpass FIR filter (0.5–40 Hz). Occular artifacts were removed using linear regression, relying on EOG channels. The AutoReject tool was employed, resulting in the removal of 17% of segments (range: 0.6–44%), interpolation of 5% of segments (range: 0.8–29%), and rejection of 23% of trials (range: 0–78%) across all TMS recordings. After applying AutoReject, the remaining data had a median of 300 trials (range: 73–300). The EEG data was then re-referenced to an average and baseline correction was applied. Finally, ICA was performed, leading to the removal of a median of 0 components (range: 0–4). Following this cleaning procedure, the evoked responses were visually inspected by two independent researchers for data quality. Only data displaying a clear evoked response and satisfied the following criteria were included: sufficiently high signal-to-noise ratio compared to baseline, no early artefactual component, no isolated sensory component (see supplementary materials: “Example data” Fig S2 for examples of trials that were included and excluded). Following this procedure, we were left with 52 TMS-evoked potentials (TEPs) (17 from wakefulness and 35 from sedation), based on a median of 300 trials (range: 73–300).

PCI^st^ was then calculated using code from Comolatti, et al.^13^. After decomposing the TMS-evoked potentials into principal components, a measure of the difference in the number of state transitions during the response compared to baseline was calculated for each component, as follows: First, amplitude distances (pointwise differences) between all timepoints were calculated for baseline and response (individually). Second, a binarization threshold $\:\epsilon\:\:$was set to maximize the (weighted) difference between the number of state transitions in the response and the number of state transitions in the base. The final measure, PCI^st^, was obtained by summing the differences between the number of state transitions across all components. PCI^st^ was used because it was more time efficient calculation-wise than PCI^LZ^ and does not require the additional step of performing source estimation of the evoked EEG. PCI^st^ has previously been reported to provide equally reliable results as PCI^LZ^^13^.

### Analysis of reports

Immediately after awakening (while propofol infusion was maintained), the participants were asked:

Q1: What did you experience?

Q2: Did you experience anything?

Because participants just awoken from anesthesia can be incoherent, hard to understand and may not always give informative answers (see Table 3 in Results), we created an *experience classification* [Strictly speaking this is a classification of the experience reports, but as the intention is to capture the underlying experience, and for the sake of brevity, we will stick with the term* experience classification*] based on a systematic evaluation of the combined evidence from both Q1 and Q2.

Responses to the first question—”What did you experience?”—were categorized based on the level of detail provided about the experience they reported (Table 1).

**Table 1:** Categorization of reports to Q1 with explanation and examples from reports.

Q1: What did you experience?		
Category	Explanation	Example
Nothing	Participants reported having no experiences	“I had no experience”
No information	Participants did not answer the question or hard to understand	No answer.
White report	Remember something but doesn’t know what	“I’m not sure…not much”
Vague report	Remember feelings/color/ faces	“Yeah, there was a … and the dog he … all the … and then they had to run, we were chased, it was very exciting”
Vivid report	Scenes and stories remembered	“I’m amazed by time - time for sex? It must be naked ladies…” “I was part of that wolf pack”

For the second question— “Did you experience anything?”—each awakening was similarly categorized into five categories (see Table 2).

**Table 2:** Categorization of reports to Q2 with explanation.

Q2: Did you experience anything?	
Category	Explanation
No	Participants positively reported having had no experience
No information	Participants did not answer the question
Maybe	Participants indicated that they were uncertain about having had experiences
Yes	Participants positively reported having had an experience

We classified the overall experience report into three classes - *no experience*, *no information* and *experience* - based on whether we considered the combined evidence from the answers to Q1 and Q2 to be suggestive of no experience, not informative, or suggestive of experience, following the scheme illustrated in Table 3 (see “Results”). This experience classification was used for the analysis of how PCI^st^ and single-channel LZc varies with reported experience prior to awakening.

### Statistical analysis

The statistical analyses were performed using SPSS^31^. In the main analysis, we assessed the relationship between experience classification (“no experience”, “no info” and “experience”) and the EEG-based measures of consciousness (PCI^st^ and 1-minute single-channel LZc) from awakenings during propofol sedation with linear mixed models (LMM). The consciousness measures were entered as dependent variables, with experience classification as a fixed effect and participant ID as a random intercept following this formula:

$$consciousness~\_measure_{{ij}} = \beta _{0} + \mathop \sum \limits_{{i = 1}}^{{k - 1}} \beta _{i} \times \exp erience\_class_{{ij}} + u_{j} + \smallint _{{ij}}$$

Here *consciousness_measure*_*ij*_ is the value for observation *i* of subject *j*,* β*_*0*_ is the intercept of the model, *Β*_*i*_ is the fixed effect coefficient, *experience_class*_*ij*_ is a categorical variable representing the experience classification, *u*_*j*_ is the random intercept for each subject, and *ε*_*ij*_ is the residual error. We report the results for the Type III F-test for the experience category fixed effect and the estimated marginal means (EMMs).

In addition, we employed a non-parametric matched-pairs Wilcoxon signed rank test to compare consciousness measures between wakefulness and sedation. For PCI^st^ we compared values recorded from wakefulness with eyes closed with the mean value of up to three sedation recordings for each participant. For single-channel LZc we compared values from wakefulness 5-minutes with eyes open and eyes closed to the mean value from up to three 1-minute sedation recordings. In addition, we compared these measures between eyes open and eyes closed conditions within wakefulness (see Figure S3 for subject-specific values for PCI^st^ and single-channel LZc across all recordings, and Figure S5 for the distribution of values across conditions). Results from the non-parametric tests are reported in more detail in the supplementary materials: “Results and discussion”.

Finally, to account for multiple comparisons, we applied the Holm-Bonferroni correction across six tests: “wake” vs. “sedation” for PCI^st^ and single-channel LZc (with both wake eyes open and wake eyes closed), and “eyes open” vs. “eyes closed” for single-channel LZc. Additionally, we tested how the classification of reports (experience, no information, and no experience) varied with PCI^st^ and single-channel LZc scores.

The significance threshold was adjusted for each test sequentially, with p-values ranked in ascending order. Tests were considered significant if their p-value met the adjusted threshold.

We assessed outliers by examining the distributions of the following: PCI^st^ values during the awake state, all PCI^st^ values under sedation, 5-minute single-channel LZc values for eyes open and eyes closed, and all 1-minute single-channel LZc values. Data points falling outside the range of 1.5 times the interquartile range (IQR) above the third quartile or below the first quartile were carefully reviewed by inspecting the cleaned data and cross-referencing with the research protocols. Values associated with poor data quality or any disruptions in the protocol were excluded.

## Results

### Participants, EEG recordings and experience reports

**Fig. 1:**
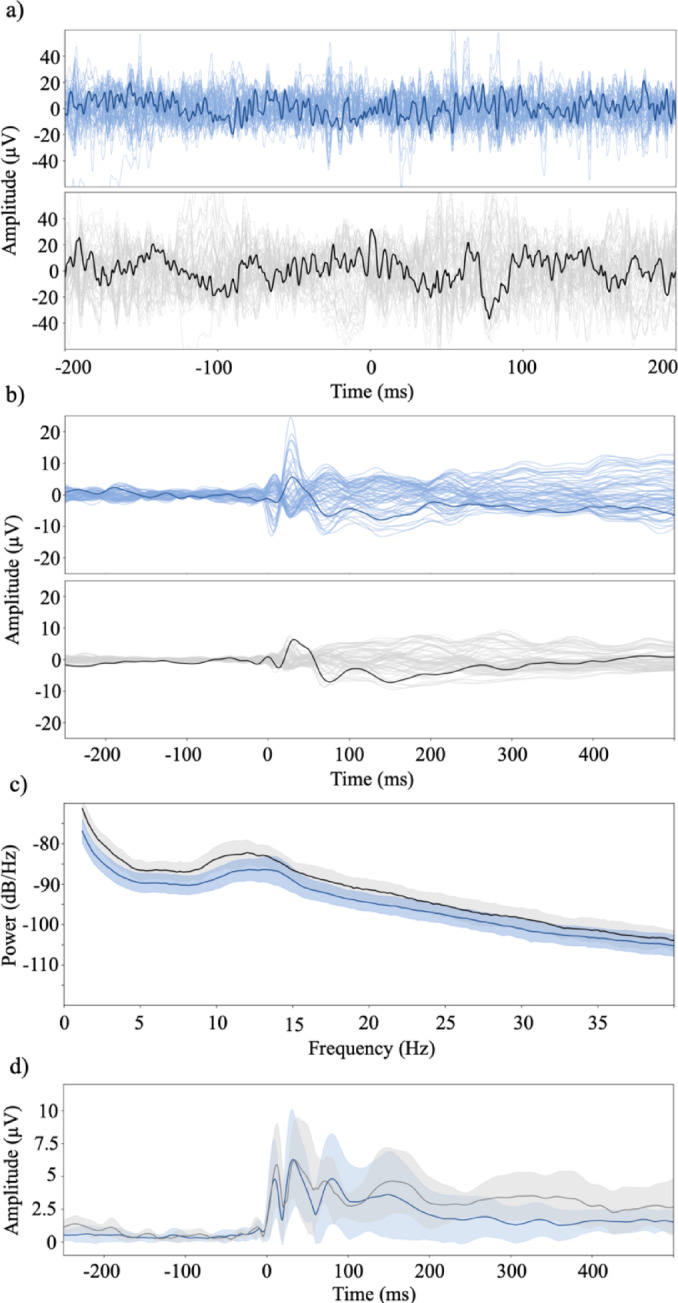
Overview of evoked and spontaneous EEG data in “Experience” (blue) and “No experience” (grey) states. The figure shows a representation of EEG data from sedation without normalizing for participant effects. Cz is highlighted in all figures. (**a**) A random epoch from spontaneous EEG recording classified as “experience” (blue) and “no experience” (grey). (**b**) A random trial from evoked EEG recording classified as “experience” (blue) and “no experience” (grey) respectively. (**c**) PSD plots of spontaneous EEG from all recordings in the “experience” (blue) and “no experience” (grey) categories respectively. Shaded areas indicate standard deviation. (**d**) Mean power plots from evoked data from all recordings in the “experience” (blue) and “no experience” (grey) category respectively. Shaded areas indicate standard deviation.

From the 20 participants that completed the study, we obtained a total of 92 recordings of spontaneous EEG: 40 from wakefulness (including eyes open and closed conditions) and 52 recordings during sedation (up to three per participants; see supplementary materials Figure S1). On the day of the experiment, three participants did not yield sufficiently high signal-to-noise ratio following the TMS pulse and we therefore only recorded spontaneous EEG data from these participants. Therefore, we only obtained a total of 55 TMS-EEG recordings: 17 from waking rest (eyes closed) and 38 from sedation recordings (up to three per participant; see supplementary materials Figure S1). Figure 1 shows examples and summaries of the EEG data used in further analysis.

Due to the time constraints during experiment, not all participants were awakened three times, and some participants were not sufficiently arousable to provide a comprehensible report. In total, the participants were awoken 52 times; 14 participants were awoken 3 times, 4 were awoken twice, and 2 were awoken only once.

The distribution of reports following the 52 awakenings is summarized in Table 3. In response to question 1 (“What did you experience?”), in a total of 28 awakenings participants either gave no report, did not awaken, or gave a report that was too incomprehensible to understand (“no info”). In 12 cases participants reported having had “vivid” experiences. In 9 cases, they reported “vague” experiences, while 2 awakenings yielded a “white” report. Thus, in total 23 out of 52 awakenings positively reported having had some form of experience (“vivid”, “vague” and “white” reports), while only one participant positively reported having had no experience in one of their awakenings (“no experience”). Immediately following the first question, participants were asked “Did you experience anything?”, with the option to answer “yes”, ”no”, or ”maybe”. In response, in 8 awakenings participants categorically stated they had no experience (“no”), 22 awakenings yielded “no info” because their responses were either absent or too vague for clear interpretation, in 4 instances they reported being unsure (“maybe”), while in 18 awakenings participants confirmed having had an experience (“yes”).

**Table 3:**
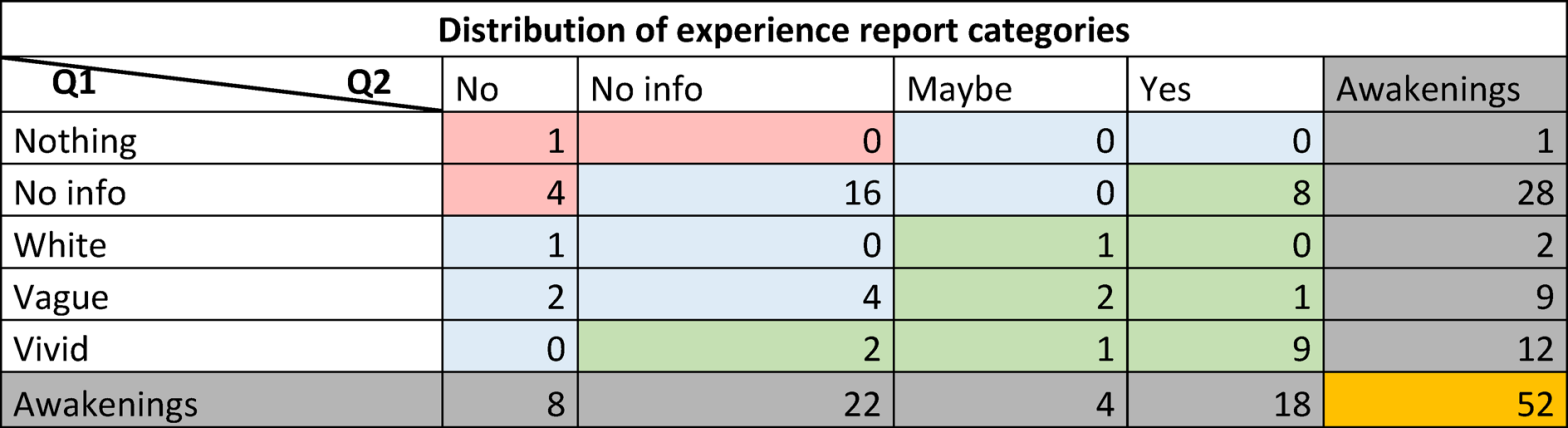
Distribution of experience report categories. The table shows numbers of awakenings in combined groups from Q1 (vertically) and Q2 (horizontally). Awakenings (greyed out) displays the total sum for each category in Q1 and Q2. Color coding represents the three classes of experience report—no experience (red), no information (blue) and experience (green).

### PCI^st^ and single-channel LZc do not vary with experience reports within sedation

Experience classification was not a significant effect in the model for how PCI^st^ varies within the sedated state (F = 0.536, *p* = 0.590). Estimated marginal means (EMMs) were higher for awakenings classified as “no experience” (M: 27.264, SD: 4.984) than for awakenings classified as either “no information” (M: 21.655, SD: 2.418) or “experience” (M: 21.704, SD: 2.664). The differences in EMMs were not significant.

**Fig. 2:**
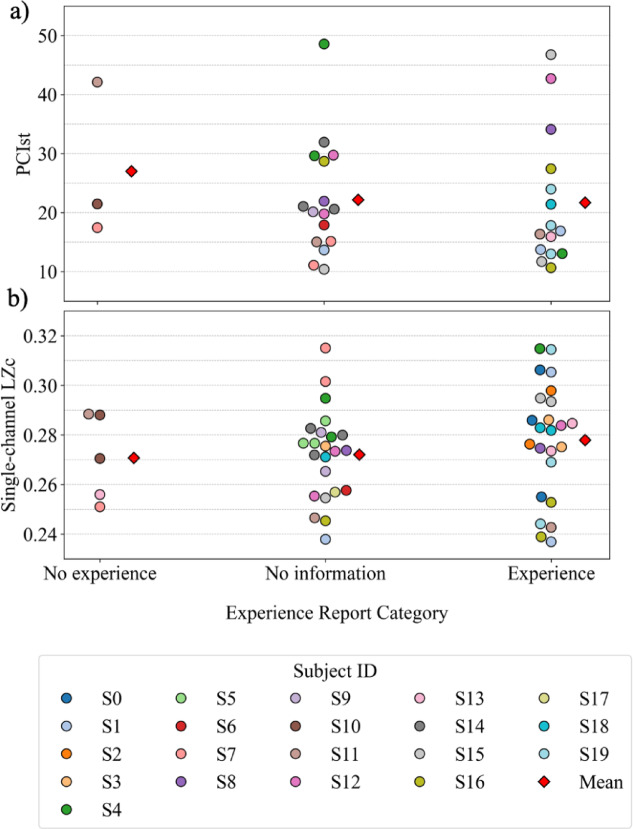
Comparison of experience categories with PCIst and single-channel LZc. The panels show the values of PCI^st^ (**a**) and single-channel LZc (**b**) plotted for each experience category. Color coding for 20 participants and mean for each category is plotted as a red diamond. The differing number of values in plots (**a**) and (**b**) results from variations in data collection between evoked EEG (**a**) and spontaneous EEG (**b**).

Figure 2 illustrates the relationship between the experience categories (x-axis) and PCIst (a) and single-channel LZc (b) (y-axis).

Experience classification was not a significant effect in the model for how single-channel LZc (F = 0.727, *p* = 0.489) varies within sedation (see supplementary materials: “Results and discussion” for similar analyses for multi-channel LZc, ACE and SCE). While the EMMs for single-channel LZc was higher for awakenings classified as “experience” (M: 0.278, SD: 0.004) than for awakenings classified as “no information” (M: 0.272, SD: 0.004), with awakenings classified as “no experience” as the lowest (M: 0.270, SD: 0.009), these differences were not significant.

### PCI^st^ and single-channel LZc decreases from wakefulness to sedation

Employing the Wilcoxon rank sign test, PCI^st^ differed significantly (*p* < 0.001) between wakefulness (M: 53.574, SD: 20.422) and sedation (the subject mean was used, across 1 to 3 awakening sessions) (M: 22.135, SD: 5.033) (see Table 4 for details).

Similarly, single-channel LZc was also lower for a mean of up to three sedation recordings (M: 0.274, SD: 0.012), compared to wake with eyes open (M: 0.325, SD: 0.020) or wake with eyes closed (M: 0.309, SD: 0.018) (see Table 4 for details). The drop in mean LZc from wake with eyes closed to the sedated state was about twice the drop from eyes open to eyes closed. See supplementary materials for similar analyses for multi-channel LZc, ACE and SCE.

**Table 4:** Wilcoxon rank-sign test between PCIst and single-channel LZc scores from wakefulness (W) and a mean computed from up to three sedation recordings (SED Mean).

Measures	State 1	State 2	Z-score	*p*-value
PCI^st^	W	SED mean	−3.41	< 0.001
Single-channel LZc	W/O	W/C	−3.62	< 0.001
	W/O	SED mean	−3.92	< 0.001
	W/C		−3.92	< 0.001

*W* state of wakefulness,* /O*  with eyes open,* /C*  with eyes closed.

Within wakefulness, single-channel LZc was lower for the eyes-closed condition (M: 0.309, SD: 0.018) than for the eyes-open condition (M: 0.325, SD: 0.020), and the difference was significant (*p* < 0.001). See supplementary materials for similar analyses of multi-channel LZc, ACE and SCE.

## Discussion

Using an intermittent awakening paradigm during propofol sedation (RASS: -4), we did not find experience classification (no experience, no information, experience) of dream reports to be a significant factor explaining variability of single-channel LZc and PCIst within deep sedation. However, our study confirms earlier findings that these measures can differentiate between wakefulness and a propofol induced sedated state. This raises the question of whether these EEG-based measures can reliably be used to track (dream) experiences within the sedated state, assuming that the participants’ immediate reports about their conscious experiences during propofol sedation can be trusted.

Traditionally, it has often been assumed (or at least stated) that propofol sedation induces a state of unconsciousness^32^. However, previous studies have also shown that humans undergoing propofol sedation often report having had experiences during the sedation^18,33^. One possible explanation as to why some report that propofol sedation is associated with no subjective experience, while other studies indicate the opposite, is the amnesic effects of propofol^34^. While an intermittent awakening paradigm has rarely been used to collect reports of experiences during sedation (but see Casey, et al.^21^), this paradigm can potentially reduce the impact of amnesia^21,34^. Here, we obtained positive reports of experience in an overwhelming majority of the awakenings with interpretable responses (82%), even though participants were deeply sedated (RASS: -4) and could only be aroused by strong physical stimulation combined with loudly calling their name. Our findings are in line with a recent study by Radek et al.^35^ which investigated the occurrence of experiences during dexmedetomidine or propofol sedation with intermittent awakenings of healthy subjects. They obtained positive reports in 90% and 74% of the awakenings from dexmedetomidine and propofol, respectively. Thus, our findings seem to support the idea that conscious experiences can frequently occur during propofol sedation, even when the individuals appear to be unconscious^32^.

Within deep sedation, experience classification was not a significant factor explaining PCI^st^ variability. However, this could be due to different causes. For one, PCI^st^ was calculated over a time period of several minutes ending one minute before awakening. It is unlikely that a report of experience in the form of dreaming reflects several minutes prior to awakening nor the whole period of recording and thus PCI^st^ may not accurately reflect the timing of the participants’ reported experiences. This complicates the comparison with the self-reported experiences. Additionally, in conditions where the presence and content of experience may fluctuate, this factor should be carefully considered and PCI^st^ may essentially not be time-resolved enough to be applied in these cases. In future experiments, one could focus on ways to investigate the temporal variation in the measures closer to the experience reports. One such attempt at achieving more time-resolved measurements is by Nieminen et al.^17^ who examined TMS-evoked responses immediately prior to awakening from sleep. They observed larger negative deflections and shorter phase-locked responses when individuals reported having had no experiences. Furthermore, they found no such difference when assessing the response from a similar number of pulses further from the time of awakening. Implicitly, this conforms to an interpretation that individuals might fluctuate between periods of rich reportable content and (nearly) no reportable content while in the sedated state. We sought to replicate these findings in our dataset in post-hoc analyses (see Figure S6 and “Differential outcomes in ITPC and GMFP analyses” in supplementary materials: “Results and discussion”). However, due to methodological differences (e.g. our stimulation site varied between individuals and notably our recording period ended 1 min prior to the awakening) we could not reasonably do so. In any case, we observed no clear variability in the TMS-evoked potentials over time.

Another technical limitation in our dataset is that the distribution of awakenings with and without dreams were heavily skewed. Compared to the awakenings in the “experience” group (*n* = 24), there were very few awakenings with interpretable reports of no experience (*n* = 5). The remaining awakenings (*n* = 23) provided no insight into the state of experience (“no information”). This draws the power of the statistical model into question and gives further ground for caution when interpreting the null finding reported here.

Finally, it is worth noting that it is particularly challenging to obtain sufficiently high quality TMS-EEG data in the context of the study. Unfortunately, sedation is associated with some agitation and movement in the process of dosage titration, induction, or during intermittent awakening, which compromises the quality of data as the soft electrode cap may shift and navigational tracking may be disturbed. This also meant that a fixed coil position (e.g. with a fixed “arm”) was unfeasible such that a researcher needed to manually control the positioning of the TMS coil. Additionally, the use of a supine position during the anesthetic period resulted in posterior clusters of EEG channels sometimes losing contact with the scalp and being difficult to correct. Thus, more EEG channels than expected had to be rejected in post-processing. Furthermore, it proved difficult to locate regions on the scalp that produced reliable evoked potentials for PCI^st^ analysis, while also reducing muscle and TMS artifacts. This resulted in significant artifacts in the TMS-EEG data. However, all TMS-EEG data were assessed by two experienced researchers, and only the datasets with a sufficient signal-to-noise ratio and no visible sensory components were included in the analysis^36^. Still, the reliability of our results may be compromised and should be considered when interpreting the findings.

Similarly, experience classification was also not a significant factor explaining single-channel LZc variability within deep sedation. This reflects a study by Aamodt et al.^19^ who also found no correlation between the presence and absence of dreaming and LZc during non-REM sleep. In contrast to PCI^st^, single-channel LZc is calculated based on a 1-minute recording of ongoing cortical activity immediately before the awakenings. However, similar to PCI^st^, the sample size for single-channel LZc is also limited, complicating the statistical test between experience classes, and a null result does not necessarily allow us to reject the hypothesis of there being a relationship. Some of the technical limitations mentioned above also apply here. Nevertheless, this finding should be taken into account when evaluating the overall evidence for whether single-channel LZc is truly able to track experiences within the same state^37–40^.

If we suppose that the EEG-based consciousness measures can reliably track the presence vs. an apparent absence of experiences of any kind, how then can we explain the drop between wakefulness and a state of sedation that is presumably filled with experiences (dreams)?

One possible explanation is that reduced values of the measures indicate the presence of experience that is somehow degraded but not absent. If we assume that consciousness can be graded, we must also then consider what we exactly mean by “graded” experience^41^. For example, experiences may be graded according to their diversity and vividness; in which case a “less conscious” individual should have less rich or vivid experience (and vice versa for a “more conscious” individual). Interestingly, we observed a drop in single-channel LZc from eyes open to eyes closed during wakefulness, reproducing previous findings (see Farnes et al.^38^). This might reflect such a gradation in experience and the measure’s sensitivity to it.

In that case, any measure that accurately reflects conscious experience should also track this gradation accurately. However, an intermittent awakening paradigm during N2 sleep was used to examine the relationship between single-channel LZc and ratings of subjective experience^19,42^. They found no robust correlation between LZc and the richness of content (as measured by “ratings of diversity and vividness of dream contents”).

An important justification for the ability of complexity measures to track consciousness is that they were designed as rough proxies for Φ (the quantitative measure associated with IIT^11^) and aim to capture the co-occurrence of integration and differentiation in the brain^43^. In theory, low complexity values correspond to low Φ^44^. However, it is possible that these measures are overly simplistic and fail to capture the finer details of the relevant kind of complexity. In this case, PCI^st^ and single-channel LZc may be too coarse proxies leaving them unable to capture structural and functional properties that are essential for consciousness.

Alternatively, if we assume individuals can fluctuate between periods of dreaming and no dreaming within the same state, the drop in these measures from wakefulness may just reflect a reduced frequency of dreaming. However, we have no evidence indicating such a fluctuation in state in the periods of sedation (see Figure S4 for time-resolved LZc values).

There were various methodological challenges in this study, which should be considered when interpreting the results presented. For one, our classification of awakenings as being associated with “experience” and “non-experience” is based on the participants’ immediate retrospective dream reports after being awoken from sedation. Thus, it is pertinent to ask whether such reports are trustworthy.

It is generally hard to conclude from dream reports whether or not participants truly had an experience before being awoken. When participants are awoken, they may be confused and can have difficulty understanding the task. They could have trouble reporting coherently (or at all), and researchers could have trouble hearing or understanding the report. Furthermore, participants may confabulate due to a positive response bias or the tendency of propofol to make people talkative. Propofol sedation is also known to impair memory, and participants may be unwilling to report for example because they lose focus or are embarrassed.

We attempted to control for these uncertainties in three ways. First, before the sedation we made sure to explicitly and repeatedly remind the participant that we were equally interested in negative reports (i.e. reports of no experience) as in positive reports, that they should report any experience they had, no matter how trivial or simple it may have seemed, that their answers would remain anonymous, and that they should merely answer the questions as honestly as possible. We did a practice run after the wake EEG recordings to ensure that the participant became familiar with the task. Second, we also included simple tests after the questions about experiences to test for confabulation and impaired short-term memory (see Table S1 in supplementary materials). For the memory test, participants were presented with 5 words and asked to repeat them, and for the confabulation test they were asked to name the country of a combination of eight real and made-up cities. The participants were able to reproduce the words presented to them and rarely fabricated answers to questions intended to confuse them. Third, and finally, we employed two questions to probe dream content; free report and a yes/no question of whether there was an experience.

In sum, we think that it seems reasonable to take the participants’ report at face value, as long as they were easily interpretable, despite the inherent uncertainty associated with subjective reports. In particular, we think that a vivid report containing stories and scenes gives reason for high confidence that the participant actually had an experience before the awakening. Given that subjective report of experience ought to be the gold standard for inferring that a human being is conscious, we should be careful of a priori discounting subjective reports due the state they report in (e.g. propofol sedation).

A final limitation worth discussing is whether or not the awakenings in this study were truly from comparable sedation states. While we did what we could to ensure the subjects were all in the same behavioral state during sedation (RASS: − 4), there are reasons to suspect that the state may have varied somewhat between awakenings and subjects. First, propofol can accumulate in fatty tissue, leading to effect site concentrations being higher in later awakenings^45^. To remedy this, the dosage was slightly reduced in some subjects after the first or second awakening, in particular after failed awakenings. Furthermore, following awakenings, the subjects are more arousable for a brief period of time. Therefore, we allowed them several minutes of quiet rest after telling them to go back to sleep before starting the recordings, assuming that they would then be back to the expected state. Finally, there was a large variation between individuals in the estimated effect site dosages required to achieve the targeted behavioral state (median: 2.0, range 0.8–3 µg/ml). This may suggest potential for differences in experiential state between individuals despite their comparable behavioral state. However, in our statistical model we attempted to control for this by defining participants as a random intercept. Thus, while it remains uncertain whether all subjects were in fact in a nearly identical state prior to awakening, we have no good reason to believe that variations in the state can explain the variation in reports observed.

In summary, we found that complexity measures were unable to discriminate between awakenings with and without dream reports. In particular, single-channel LZc values — computed from data gathered immediately preceding the report — were not different between awakenings with and without dreams. While the same is true for the PCI^st^ values, they were computed from a period further separated from the awakening, and as an aggregate of a 10-minute period, making their direct connection to reports more dubious. While there are technical limitations to the present study, both complexity measures computed from the sedated state largely overlapped with values typically associated with “unconscious” states, despite frequent reports of dream-like experiences in most of the awakenings. Considering that many states traditionally characterized as “unconscious” have been shown to involve experiences^32^ it may be relevant to re-evaluate how low complexity values should be interpreted with regards to classifying states of consciousness. At the very least, we should refrain from referring to states associated with low complexity values as unconscious.

## Electronic supplementary material

Below is the link to the electronic supplementary material.

Supplementary Material 1

## Data Availability

The raw EEG data is publicly available at OpenNeuro (doi:10.18112/openneuro.ds005620.v1.0.0) and has been organized according to the Brain Imaging Data Structure (BIDS) format^46,47^.
