# Supplementary material for: A repeated awakening study exploring the capacity of complexity measures to capture dreaming during propofol sedation
Source: Sci Rep. 2025 Sep 24;15:32746. doi: 10.1038/s41598-025-12695-z (PMC12460656; doi:10.1038/s41598-025-12695-z)
Supplement: Supplementary file 1 — Supplementary Material 1 [file 41598_2025_12695_MOESM1_ESM.docx]

Supplementary materials

| **Questions upon awakenings** |
| --- |
| Q1: Did you experience anything? |
| Q2: What did you experience? |
| Q3: Remember these five words: [five one-syllable common nouns]. |
| Q4: I will name six cities. Answer the country if you know, answer ‘next’  otherwise: [some well-known and made-up cities] |
| **Table S1: Questions asked upon awakening** |

| a) | b) |
| --- | --- |
| **Figure S1:** Distribution of awakenings for evoked data (a) and spontanuous data (b) classified as Experience, No information and No experience among all recorded files that were included in the analysis. “Excluded” here represents the number of files that were excluded in the analysis. | |

| a) | b) |
| --- | --- |
| **Table S2:** Summary of preprocessing statistics for spontaneous data (a) and evoked data (b). | |

| 1. Included |
| --- |
| 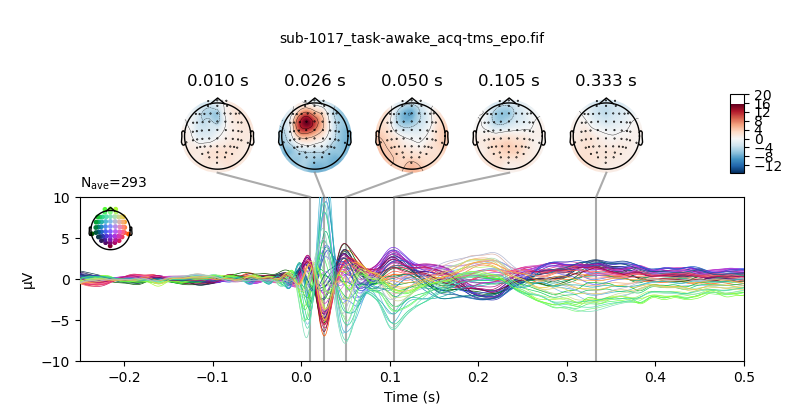 |
| 1. Excluded |
| 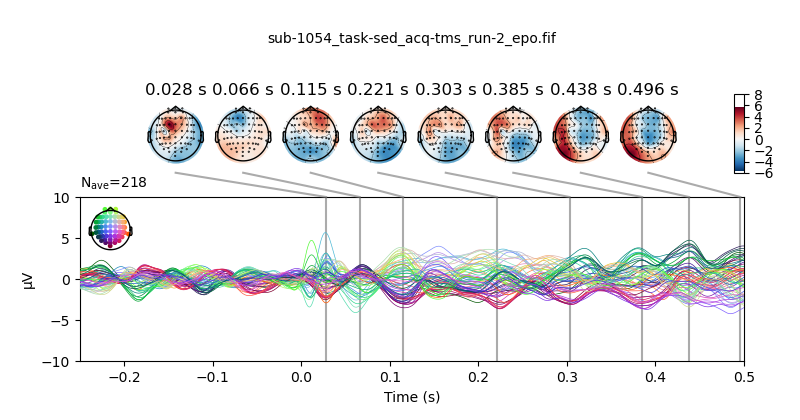 |
| **Figure S2: Example of TMS evoked response** from trials that were included (a) and excluded (b). |

| 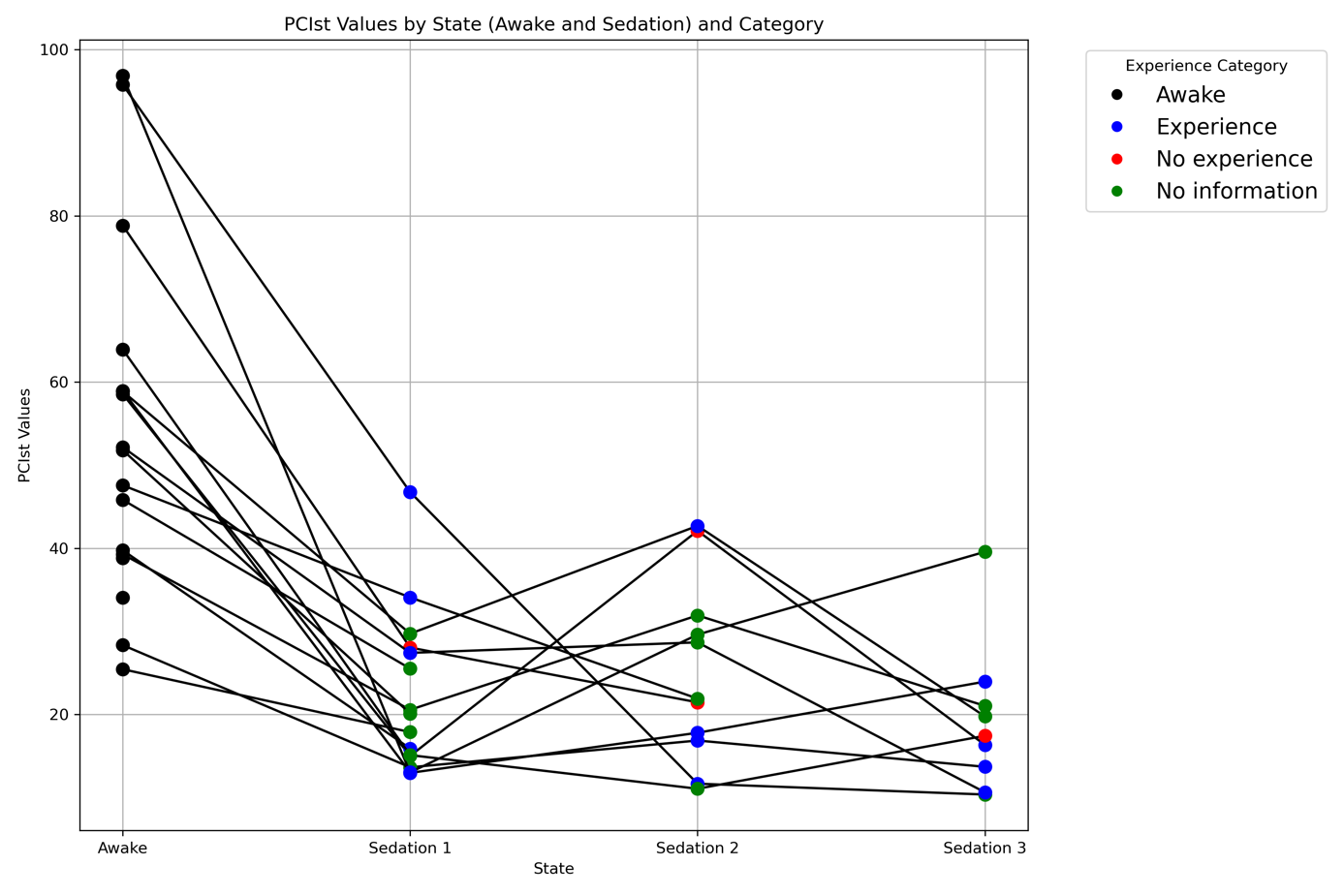 |
| --- |
| 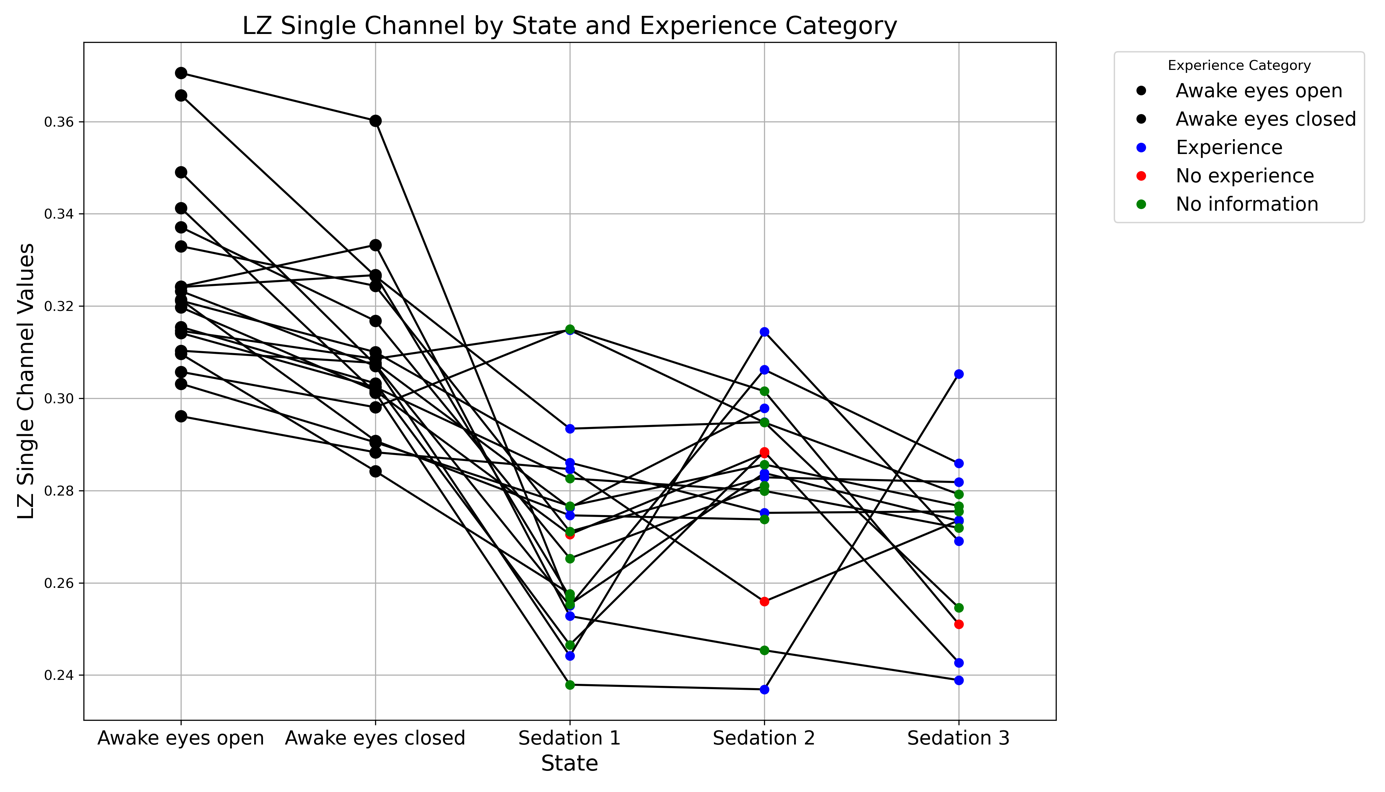 |
| **Figure S3: Longitudinal Values of PCIst (top) and Single-Channel LZc (bottom)** across wakefulness and up to three sedation recordings. The color coding indicates experience classification: Blue for awakenings classified as *Experience*, Red for *No experience*, and Green for *No information*. |

| 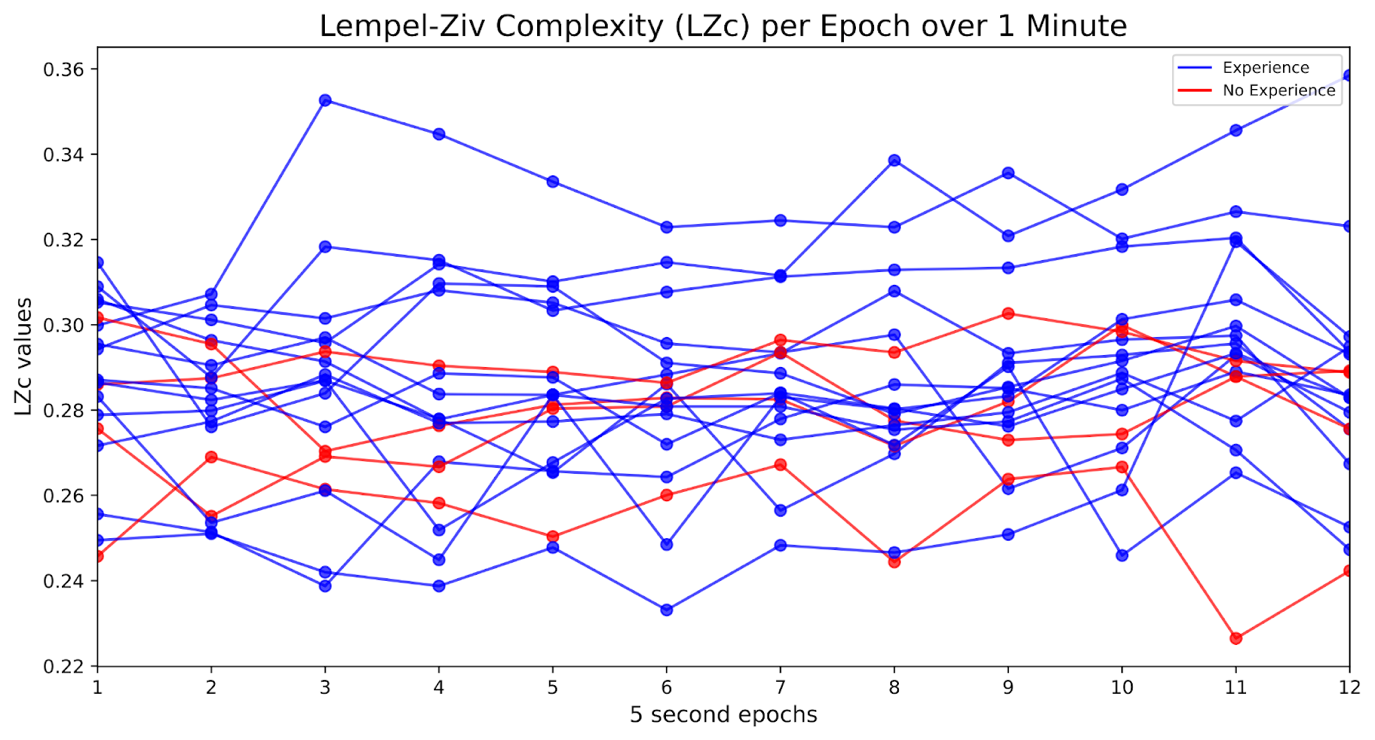 |
| --- |
| **Figure S4:** LZc values calculated for each 5-second epoch in a 1-minute recording |

# Methods

Based on the evoked TMS data we also calculated the intertrial phase clustering (ITPC) in the alpha band for the first and last 15 and all trials to determine the length of the phase locked response. ITPC data was preprocessed like the PCI data and then band-pass filtered for the alpha band (8-13 Hz). We looked at the alpha band because a recent study by Nieminen et al. (2016), showed a larger negative deflection and shorter phase-locked response when subjects reported having had no experiences compared to when they reported having had experiences. ITPC data was then used to determine the length of phase clustering for the evoked response and defined as the time point when phase-locking dropped to zero.

We also calculated, for all frequency bands, the global mean field power (GMFP) from the evoked data for the first and last 15 and all trials. Results from Nieminen et al. (2016) displayed a correlation between the amplitude of the negative evoked deflection and the length of dream report. Since our data represented evoked data from different sources on the scalp, we chose GMFP as a similar measure.

**ITPC (on alpha band) and GMFP (all bands) calculations from evoked data**

Groups of “experience” and “no experience” underwent comparative analysis using evoked TMS-EEG data and following a similar approach outlined in Nieminen et al. (2016). Initially, the preprocessed EEG data was bandpass filtered in the alpha frequency band. For each channel in every TMS-EEG file associated with an awakening, we calculated the duration of phase locking post-TMS pulse. Phase locking values below the significance threshold of alpha = 0.05 were reset to 0. The endpoint of phase locking, termed 'ITPC_drop', was determined as the first instance when the Inter-Trial Phase Coherence (ITPC) dropped to zero.

To enhance data reliability, we retained “itpc drop” values for channels that exceeded a 15 ms duration post-TMS pulse start and that occurred no later than 15 ms prior to the epoch's end at 500 ms. This step was necessary for excluding channels with low signal-to-noise ratios. Subsequently, we calculated the average “itpc drop” values for each group; “no experience” and “experience”. To assess differences in ITPC drop between the groups, we employed permutation statistics. From the combined dataset of both groups, we generated 10,000 randomized samples. These samples were used to compute new ITPC differences, contrasting them against the original phase-locked durations, thus producing a one-tailed p-value.

In an effort to analyze our data as similarly we could to Nieminen et al. (2016), we calculated the global mean field power (GMFP). To do this we first averaged the data across all epochs to get the mean EEG signal for each channel across epochs. We then took the absolute value of this mean and averaged it across channels. We did this approach for all epochs and the first and last 15 epochs.

We then employed the Wilcoxon rank sum test to analyze the GMFP data at each individual time point between groups of “no experience” and “experience”. For each comparison, we computed p-values to test the statistical significance of the observed differences between groups of “experience” to “no experience”.

**Multi-channel LZc, ACE and SCE calculations from resting state preprocessed data**

Multi-channel LZc was computed similar to the approach outlined in the main text, with the distinction being its treatment of the EEG data as a whole. Specifically, the EEG dataset was treated as a binary matrix, and the LZc complexity was calculated across all channels.

SCE and ACE are both measures of the diversity of neural activity in a population of neurons. They are also calculated by binarizing the EEG data like the scheme for LZc. SCE is calculated by measuring the diversity among a subsets or coalitions of channels that are in phase. ACE is similarly calculated by measuring the diversity among coalitions of channels that are active.

**Statistical analysis of multi-channel LZc, ACE and SCE**

Similar to our approach for PCIst and single-channel LZc, we individually employed a linear mixed model with multi-channel LZc, ACE or SCE as a dependent variable, report category as a fixed effect and participant ID as a random intercept. We report the overall results from the analysis (F-score and associated p-value).

We also used a non-parametric matched pairs Wilcoxon signed rank test to individually compare these measures between eyes open and eyes closed scores from wakefulness with the mean value from up to three sedation recordings. Additionally, we compared the measures between eyes open and eyes closed condition within wakefulness.

# Results and discussion

## Sedation decreases measures of complexity and signal diversity.

We compared how brain complexity through PCIst and signal diversity through single- and multi-channel LZc, SCE and ACE varied in propofol sedation compared to wake state. For all measures we saw a considerable decrease in measure values from wakefulness to a mean calculated from sedation scores (see Table S3). This was true regardless of whether the wake condition being compared involved eyes closed or eyes open. We also saw a decrease from wakefulness eyes open and eyes closed conditions for all measures(see Table S3).

| Measures | State 1 | State 2 | Z-score | p-value |
| --- | --- | --- | --- | --- |
| PCIst | W | SED Mean | -3.41 | <0.001 |
| Single-channel LZc | W/O | W/C | -3.62 | <0.001 |
|  | W/O | SED Mean | -3.92 | <0.001 |
|  | W/C |  | -3.92 | <0.001 |
| Multi-channel LZc | W/O | W/C | -2.58 | 0.010 |
|  | W/O | SED Mean | -3.62 | <0.001 |
|  | W/C |  | -3.285 | 0.001 |
| ACE | W/O | W/C | -3.55 | <0.001 |
|  | W/O | SED Mean | -3.92 | <0.001 |
|  | W/C |  | -3.92 | <0.001 |
| SCE | W/O | W/C | -3.62 | <0.001 |
|  | W/O | SED Mean | -3.92 | <0.001 |
|  | W/C |  | -3.92 | <0.001 |
| Table S3: Wilcoxon rank-sign test between PCIst, single and multi-channel LZc, ACE and SCE scores from wakefulness (W) and a mean computed from up to three sedation recordings (SED Mean). W = state of wakefulness, /O = with eyes open, /C = with eyes closed. | | | | |

Figure S5 illustrate the downward trend of complexity and diversity measures’ values from awake to sedation recordings. For the third sedation recordings we had fewer data points.

| 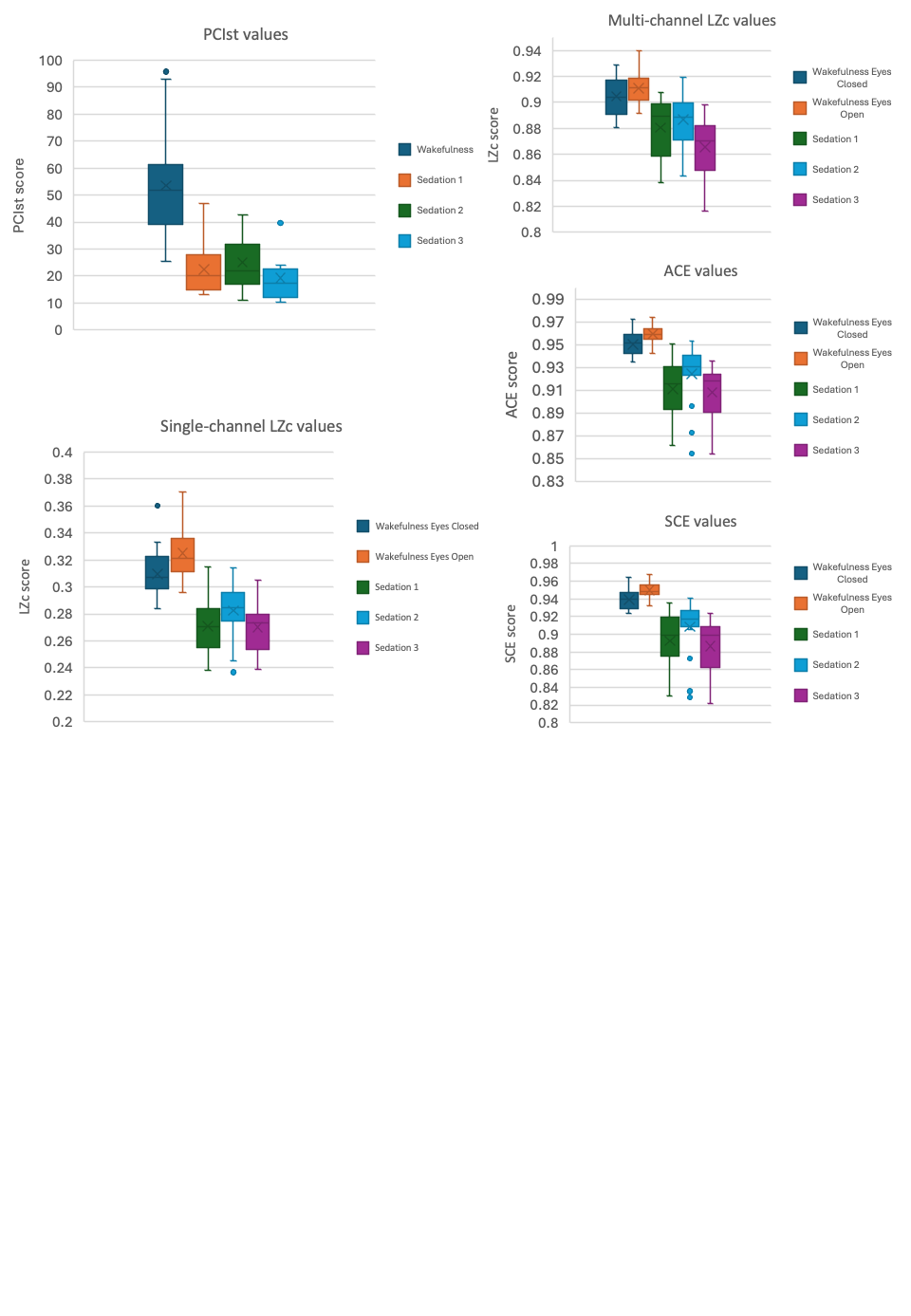**Figure S5: Boxplots of all values in all conditions** from PCIst, single- and multi-channel LZc, ACE and SCE. Awake – Eyes closed: Wake condition with eyes closed, Awake – eyes open: wake condition with eyes open, Sed 1-3: first to third awakening in the sedation condition. |
| --- |

**Multi-channel LZc, ACE and SCE does not vary with experience reports within sedation**
 Diversity measures did not vary with experience reports within sedation state upon conducting LMMs for multi-channel LZc (F = 0.078, p = 0.925), ACE (F = 0.491, p = 0.615) or SCE (F = 0.544, p = 0.584).

**Differential outcomes in ITPC and GMFP analyses**

Our analysis of Inter-Trial Phase Coherence (ITPC) revealed differences between the “no-experience” and “experience” groups, specifically in the first 15 trials. However, this distinction was not evident when considering the entire range of epochs or the last 15 trials. The observations should not be considered meaningful as there is little reason to suspect that the first 15 trials of 10-minute recordings to be representative of experiences reported compared to all or last 15 trials.

In our Global Mean Field Power (GMFP) analysis, we observed no considerable differences between the two groups, regardless of whether we examined the first 15, last 15, or the entire set of epochs.

Fig S6a shows the distribution of ITPC values for the “no experience” and “experience” categories at timepoints where they dropped to zero and the number of channels for each time interval. Fig S6c shows a comparison of the logarithm of mean GMFP between the “no experience” and “experience” categories.

Fig S6b shows mean PCIst values with standard error and results of a two-sided t-test for increasing window lengths.

| 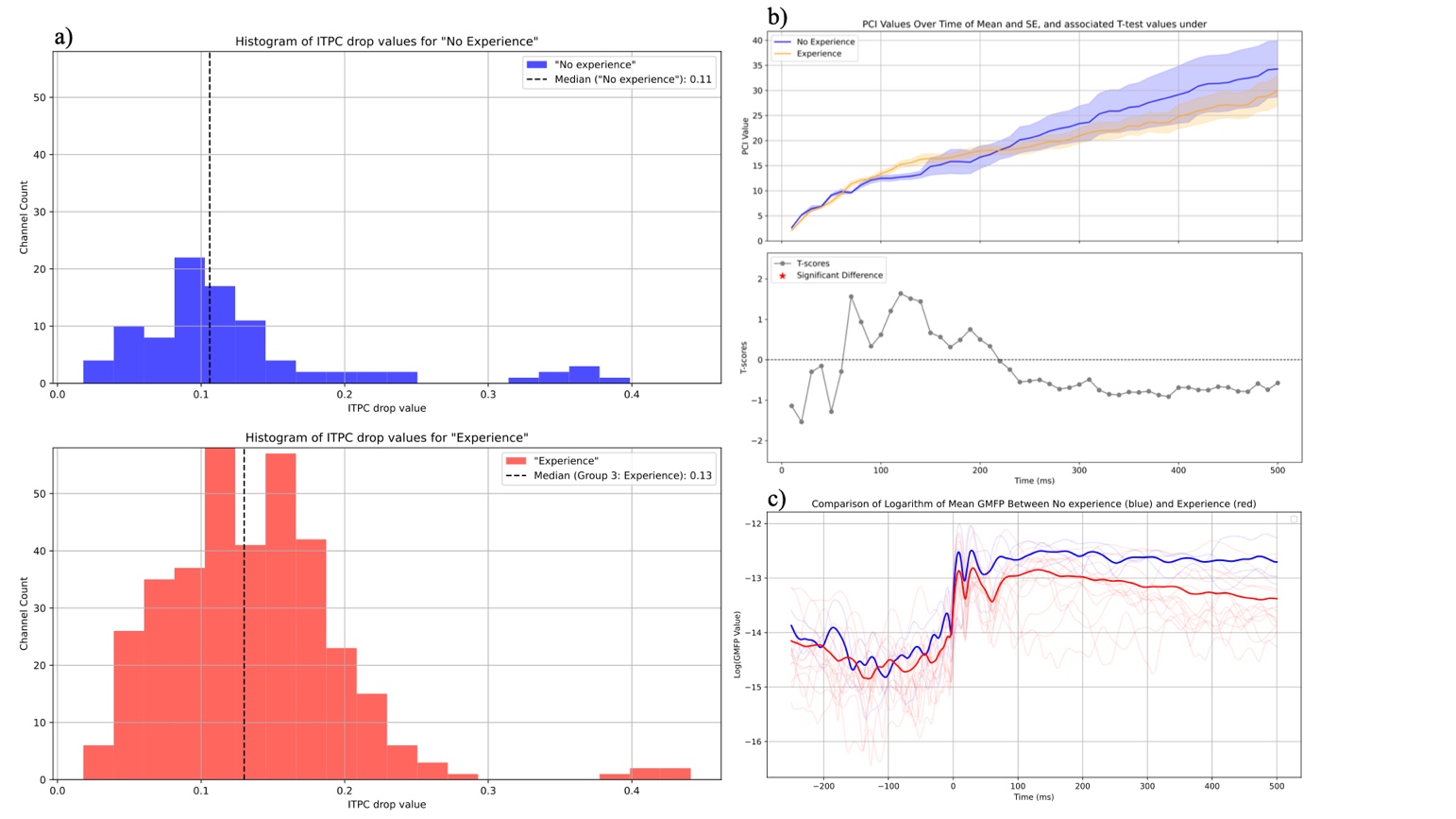 |
| --- |
| **Figure S6: Inter trial phase clustering (ITPC) drop values, PCIst values for different window lengths and global mean field power (GMFP).** a) Histograms of ITPC values when ITPC dropped to zero (in seconds) and number of channels for each ITPC drop value, upper histogram (blue) = No experience, lower histogram (red) = Experience, b) Mean PCIst values and standard error calculated for increasing window lengths (x-axis), experience (orange) and no experience (blue). Beneath is the result of two-sided t-test between the experience categories for each window length. A red star would have marked a significant result. The PCIst values were not significantly different for any timescale, even with no correction for multiple comparisons. c) comparison of the logarithm of mean GMFP between No experience (blue) and Experience (red). |
